# Errare machinale est: the use of error-related potentials in brain-machine interfaces

**DOI:** 10.3389/fnins.2014.00208

**Published:** 2014-07-22

**Authors:** Ricardo Chavarriaga, Aleksander Sobolewski, José del R. Millán

**Affiliations:** Defitech Chair in Non-Invasive Brain-Machine Interface, Center for Neuroprosthetics, School of Engineering, Ecole Polytechnique Fédérale de LausanneLausanne, Switzerland

**Keywords:** brain-machine interface, error-related potentials, reinforcement learning, EEG, neuroprosthesis, hybrid BCI

## Abstract

The ability to recognize errors is crucial for efficient behavior. Numerous studies have identified electrophysiological correlates of error recognition in the human brain (error-related potentials, ErrPs). Consequently, it has been proposed to use these signals to improve human-computer interaction (HCI) or brain-machine interfacing (BMI). Here, we present a review of over a decade of developments toward this goal. This body of work provides consistent evidence that ErrPs can be successfully detected on a single-trial basis, and that they can be effectively used in both HCI and BMI applications. We first describe the ErrP phenomenon and follow up with an analysis of different strategies to increase the robustness of a system by incorporating single-trial ErrP recognition, either by correcting the machine's actions or by providing means for its error-based adaptation. These approaches can be applied both when the user employs traditional HCI input devices or in combination with another BMI channel. Finally, we discuss the current challenges that have to be overcome in order to fully integrate ErrPs into practical applications. This includes, in particular, the characterization of such signals during real(istic) applications, as well as the possibility of extracting richer information from them, going beyond the time-locked decoding that dominates current approaches.

## 1. Introduction

Errare humanum est, perseverare autem diabolicum

–Seneca the younger

**Figure 1:**
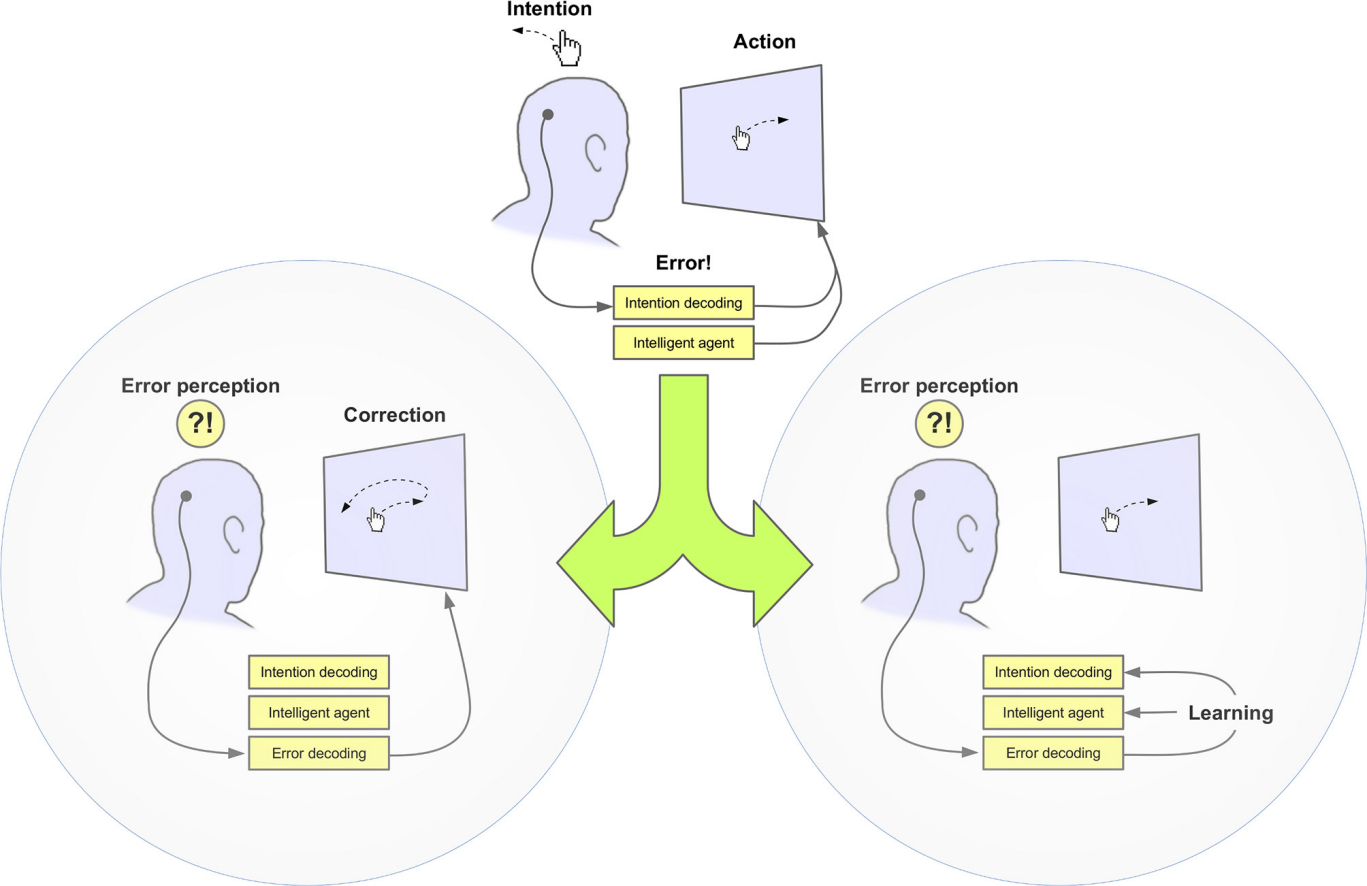
**Exploitation of error-related potentials to improve BMI performance. Left:** Single trial detection of these potentials, indicating erroneous actions, is used to trigger a corrective action, e.g., preventing execution of the last BMI command. **Right:** ErrPs are used to update the BMI classifier or the device controller by means of reinforcement learning.

The ability of human and non -human animals to learn and adapt their behavior is largely based on their capacity of identifying erroneous actions (Rabbitt, 1966). Several studies have reported that such events elicit distinct neural responses, which can be observed using different neuroimaging techniques including fMRI, scalp and intracranial electroencephalography (EEG), and magnetoencephalography (MEG). In particular, it has been demonstrated that the electrophysiological signatures of this error processing—i.e., error-related potentials, ErrPs– can be reliably decoded on a single-trial basis, thus allowing their use through brain-machine interface (BMI) systems as a means to improve the machine's performance, similarly to animals. For instance, typically BMIs aim at decoding user's intentions from the neural activity (e.g., as recorded by EEG). Misclassification of these intentions results in an erroneous command. The user's subsequent perception of such error can elicit an ErrP and the successful decoding of this response would allow the system to take corrective actions, e.g., by preventing the erroneous command from being fully executed or reverting its outcome (Schalk et al., 2000; Ferrez and Millán, 2008a; Dal Seno et al., 2010). Alternatively, ErrPs can be used to reduce the possibility of the error reappearing in the future through re-calibration of the system, allowing it to “learn from its mistakes” (Artusi et al., 2011; Llera et al., 2011). These approaches are illustrated in Figure 1. They combine the decoding of one brain signal (e.g., correlates of motor imagery or stimulus recognition) for controlling the device and the ErrP as a corrective mechanism, thus corresponding to hybrid BMI systems (Pfurtscheller et al., 2010). Notably, the same principles can also be applied to human-computer interaction (HCI) systems when input devices other than BMI are employed (Parra et al., 2003; Chavarriaga and Millán, 2010; Wang et al., 2011; Zander and Kothe, 2011; Zander and Jatzev, 2012). Interestingly, these ErrPs are naturally elicited during human interaction with the machine. This means that information about the user's cognitive assessment of such interaction can be obtained implicitly, without a need for training or asking the users to actively generate them. Systems that decode this information are sometimes referred to as *passive* BMIs; as opposed to the so-called *active* BMIs where the brain signals are consciously modulated by the user to control a given device or application (Zander and Kothe, 2011). However, caution should be taken not to interpret this as if the user played an entirely passive role during the interaction. In fact, ErrPs have been shown to be modulated by the user's level of engagement in the task (Hajcak et al., 2005).

In the last decade, researchers have provided ample evidence of the feasibility of such approaches. Here we review this work, starting with a short description of different error-related electrophysiological patterns (section 2). For more detailed account of the neural basis of these signals, readers can refer to reviews by Taylor et al. (2007); Hoffmann and Falkenstein (2012); Wessel (2012), and Ullsperger et al. (2014). Here we focus on signals that have been mostly exploited for brain-machine interfacing and primarily discuss electroencephalographic signals found using non-invasive recording techniques (section 3). We go on to present different strategies that can be applied to increase the robustness of the BMI system by incorporating single-trial ErrP recognition in both able-bodied subjects and users with motor disabilities (sections 4 and 5). We also present recent efforts to integrate these signals into real-world applications (section 6). Finally, we review the techniques used for decoding these potentials (section 7) and discuss current challenges in the study and exploitation of these signals (section 8).

## 2. Error-related brain activity

Early reports of error-related brain activity date back to the early 1990's (Falkenstein et al., 1991; Gehring et al., 1993). These studies showed a characteristic EEG event-related potential (ERP) elicited after subjects committed errors in a speed response choice task. This pattern is characterized by a negative potential deflection, termed the *error-related negativity* (ERN), appearing over fronto-central scalp areas at about 50–100 ms after a subject's erroneous response (Falkenstein et al., 2000). This negative component is followed by a centro-parietal positive deflection (Pe). Modulations of this latter component have been linked to the subject's awareness of the error. Interestingly, correlations have been found between the ERNs and behavioral adjustments following these errors, e.g., post-error response slowing (Debener et al., 2005; Frank et al., 2005; Themanson et al., 2012); supporting the idea that the signal indeed reflects an action monitoring process (Holroyd and Coles, 2002). This is further corroborated by the fact that the ERN amplitude seems to be modulated by the importance of errors in the given task (Frank et al., 2005; Taylor et al., 2007), as well as the subjective awareness of the error (Falkenstein et al., 2000; Wessel, 2012; Navarro-Cebrian et al., 2013). Regardless of such functional modulations, these signals are also influenced by individual differences and certain pathological conditions (Olvet and Hajcak, 2008). Importantly, however, these signals have been shown to be quite reliable over time (Olvet and Hajcak, 2009) and across different tasks (Riesel et al., 2013).

A similar medial-frontal EEG pattern has been reported to appear after presentation of “feedback,” i.e., the delayed result of a choice or action. This *feedback-related negativity* (FRN), appearing between 200 and 300 ms after feedback onset, is modulated by choices leading to losing situations in strategic gambling tasks (Cohen et al., 2007), as well as subject-specific sensitivity to reinforcement signals (Frank et al., 2005). Interestingly, similar signals are also elicited in the absence of motor response or while observing errors committed by a different person or agent (van Schie et al., 2004; Yeung et al., 2005; Zander et al., 2008; Chavarriaga and Millán, 2010; Zander and Jatzev, 2012). Mounting evidence provides further support of the link between these signals and reward or utility prediction errors, suggesting that ErrPs are generated when the actual outcome does not correspond to the expected one (Holroyd and Coles, 2002; Holroyd et al., 2003; Nieuwenhuis et al., 2004; Yeung et al., 2005). Such information can be used for learning by adjusting the behavior to minimize errors, as proposed by the reinforcement learning theory (Sutton and Barto, 1998).

It is worth to notice that, although these signals are typically referred to as “negativities,” the EEG correlates of performance monitoring comprise a uniform sequence of ERP components irrespective of the error source (Ullsperger et al., 2014). These include the fronto-central negative deflections related above, followed by a fronto-central positive deflection and then a later parietal positivity. This pattern is found after self-generated errors (i.e., the ERN/Pe complex), stimulus presentation (i.e., N2/P3 complex), and feedback errors (i.e., FRN/P3 complex). It is not entirely clear to what extent these signals share common underlying processes. Several studies using fMRI, EEG-based source localization, and intra-cranial recordings suggest that the fronto-central ERP modulations commonly involve the medial-frontal cortex, specifically the anterior cingulate cortex (ACC) (Ullsperger and von Cramon, 2001; Brázdil et al., 2002; van Veen and Carter, 2002; Herrmann et al., 2004; Taylor et al., 2007).

Lastly, ErrPs seen as distinct patterns in the temporal domain of electrophysiological signals are not an exhaustive description of the observable EEG phenomena. Accumulating invasive and non-invasive studies are also demonstrating frequency modulations, specifically with erroneous responses eliciting an increase of theta activity followed by a decrease of beta rhythm amplitude (Trujillo and Allen, 2007; Cohen et al., 2008; Koelewijn et al., 2008; Cavanagh et al., 2009, 2012). Moreover, connectivity studies reveal patterns of cross-regional synchronizations, pointing to influences from ACC to prefrontal areas (Cavanagh et al., 2009).

**Figure 2:**
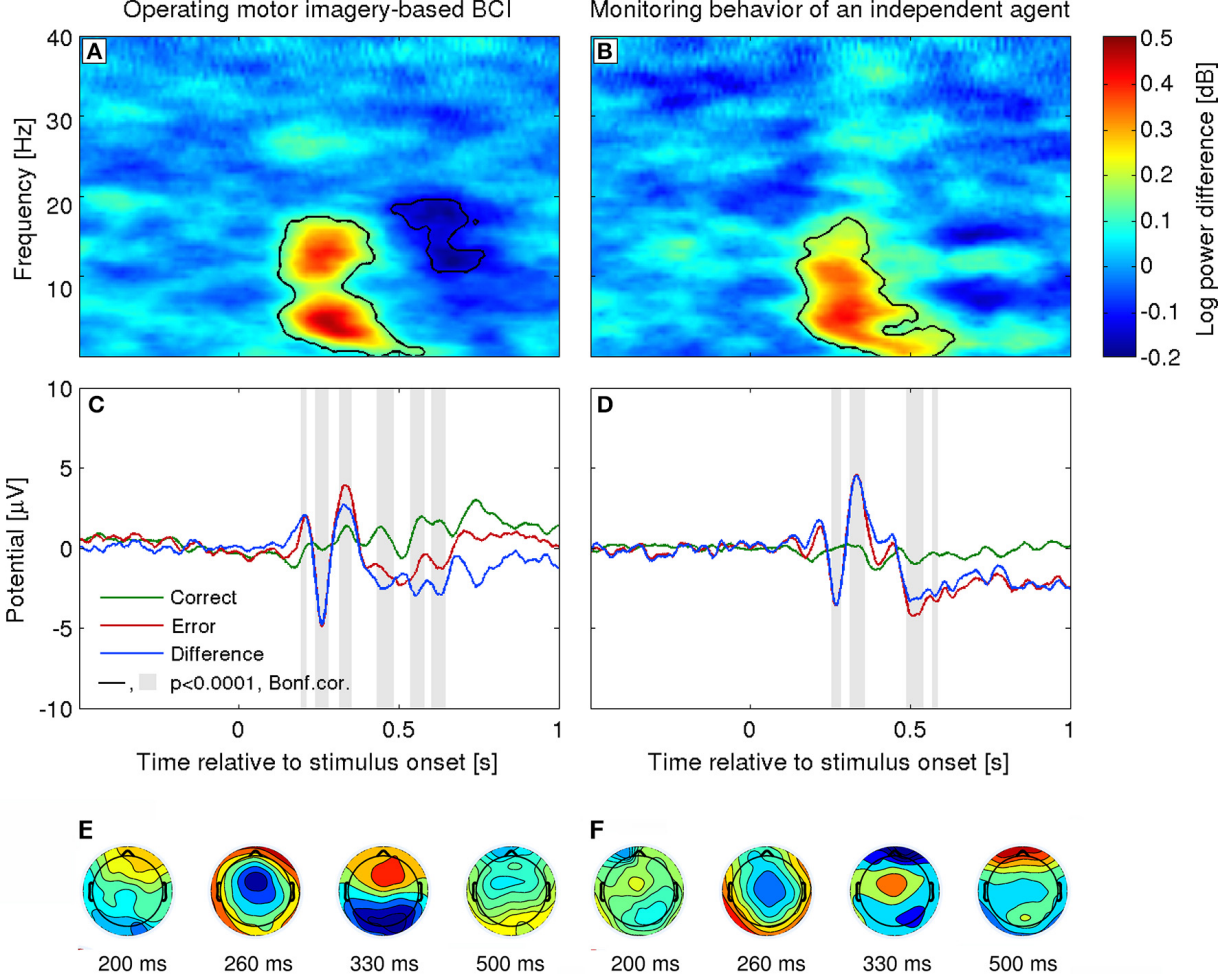
**Error-related potentials in a 2-class task used in BMI**. Left column, Interaction ErrP. The cursor movement is controlled by a MI-based BMI (Ferrez and Millán, 2008a). Right column, Monitoring ErrP: The cursor moves automatically and the user is asked to evaluate whether it moves toward the target location (Chavarriaga and Millán, 2010). **(A,B)** Event-related spectral perturbation. **(C,D)** Grand-average ERP at electrode FCz for correct, error and difference (error minus correct) conditions. *t* = 0 corresponds to the stimulus presentation onset (i.e., cursor movement). **(E,F)** Topographical representation of the group average difference ERP for both the interaction (*N* = 4) and monitoring paradigms (*N* = 6). Activity is color coded from blue to red corresponding to the range [−5 5] uV.

As already mentioned, several studies have reported an evoked response to errors in the user intention decoding when using BMI systems (c.f., Figure 2). This response exhibits the same pattern of modulations as described above. The difference waveform (error minus correct) over fronto-central areas is characterized by an initial positive peak at about 200 ms after feedback presentation, followed by a larger negative deflection at about 250 ms and a third larger positive peak at about 320 ms. Furthermore, estimation of the intracranial activity using sLoreta (Pascual-Marqui, 2002) indicated that the signals elicited during brain-machine interaction were generated in the ACC, consistent with other error-related EEG correlates (Ferrez and Millán, 2008a; Lopez-Larraz et al., 2010; Iturrate et al., 2013a). Notably, the term *error-related potential* (ErrP), has since become quite widespread within the BMI community, covering electrophysiological responses elicited in a number of paradigms. Resolving its relationship to ERP components, and their functional modulations, typically identified in basic cognitive neurosciences is beyond the scope of this BMI-focused review (although we refer to one relevant confound below). It can be considered as a useful umbrella term for application-driven research, albeit to a certain extent at the cost of correspondence with fundamental investigations. This is, however, partly justified by different research settings: closed-loop usability and practicalities of single-trial decoding are rarely the chief concern of basic neuroscience, while the latter's typical abstract, distilled experimental paradigms are not employed by engineering-oriented researchers.

## 3. Error-related potentials for BMI

**Figure 3:**
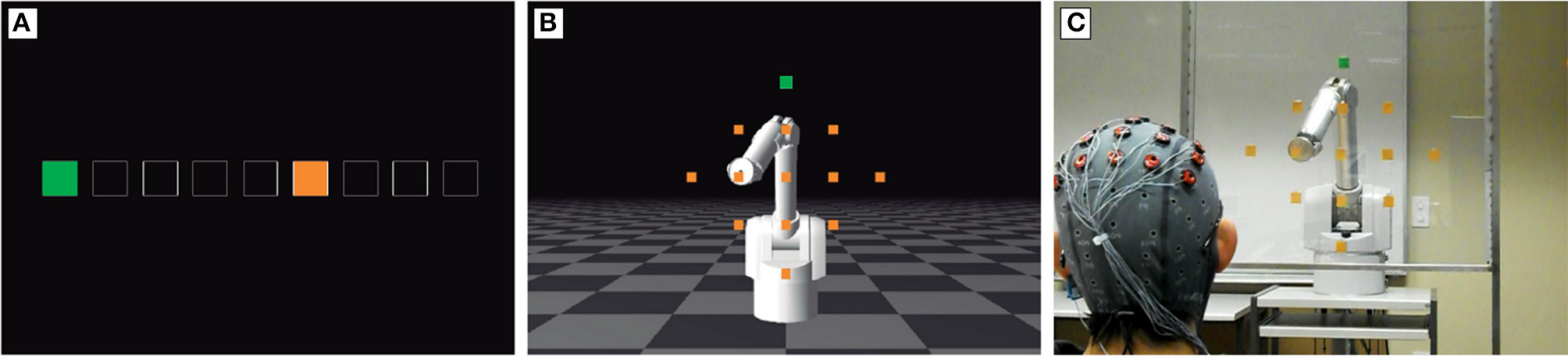
**Several experimental protocols used to study ErrPs during brain-machine interaction. (A)** 1-D cursor control (Ferrez and Millán, 2008a; Chavarriaga and Millán, 2010; Tsoneva et al., 2010; Goel et al., 2011; Zhang et al., 2012; Iturrate et al., 2013a, 2014). The cursor (orange square) moves in discrete steps toward a target location (green square). **(B,C)** 2-D control of a simulated and real robotic arm (Omedes et al., 2013; Iturrate et al., 2014).

Following the basic neurophysiological findings described in the preceding section, several studies aimed at assessing whether similar signals were also to be found when the errors were produced by a machine as a result of a misclassification of the user's intention while operating an actual or simulated BMI. In a first report (Schalk et al., 2000) showed in four healthy subjects that ErrPs are elicited at the end of erroneous trials when they controlled a 1-D cursor using a non-invasive BMI based on modulation of mu and beta EEG rhythms. This approach was further developed by Ferrez and Millán (2008a) in a study on five subjects using a 2-class motor-imagery (MI) based BMI controlling a cursor moving in discrete steps (c.f., Figure 3A). They showed that the ERPs elicited after each command could be decoded as corresponding to the error or correct condition with an accuracy of about 80%. Simultaneously, other studies tested the feasibility of decoding the error-related activity elicited after manual responses (Blankertz et al., 2003; Parra et al., 2003).

Further studies demonstrated other encouraging features of ErrPs for their use in BMI applications. Firstly, as with the ERN, they have been shown to be quite stable over time. ErrP classifiers maintained the same performance when tested several months after their calibration (Ferrez and Millán, 2008a; Chavarriaga and Millán, 2010). Furthermore, these signals seem to be mainly related to a general error-monitoring process, instead of specificities of the particular task that was performed. Iturrate et al. (2014) compared ErrPs elicited in three tasks in which subjects (*N* = 6) monitored the operation of devices of different degree of complexity: a 1-D cursor movement (Figure 3A), and a simulated (Figure 3B) and real robots (Figure 3C) moving in a 2-D space. Their results show that ErrPs across these tasks significantly differ in the latency of the peak modulations, but not in amplitude or overall waveform, thus suggesting the possibility of identifying task-independent markers of erroneous brain-machine interaction. Along the same lines, similar waveforms have been reported in tasks using different feedback modalities (Lehne et al., 2009; Perrin et al., 2010; López-Larraz et al., 2011; Chavarriaga et al., 2012).

An account of ErrP usage in BMI applications must indicate several specific confounds these signals may be susceptible to. Since –due to the nature of BMI– such applications often involve moving stimuli, there is the possibility that observed signals may be contaminated with electrooculographic (EOG) artifacts due to eye movements. This can bias the decoding particularly in application designs where direction of feedback movement is related to correctness of action. To counter this confound, researchers –especially in in proof-of-concept studies– may take care to ensure that the location or movement of target stimuli are balanced. Fortunately, it has consistently been found that ocular artifacts have little influence on the signals used for the decoding (Ferrez and Millán, 2008a; Chavarriaga and Millán, 2010; Iturrate et al., 2010; Artusi et al., 2011; Spüler et al., 2012): Nevertheless EOG artifacts remain an ever-present concern in EEG studies using moving stimuli and their potential impact should be systematically assessed.

A different possible confound is that the observed potentials are more related to the rarity of the erroneous events than their valence. To evaluate this, Ferrez and Millán (2008a) and Chavarriaga and Millán (2010) performed experiments with error rates of 50% and 40%, respectively. In both cases they report similar ErrPs than those obtained with lower error rates, although with lower amplitudes. Similarly, the N200/P300, as well as the FRN signals have also been reported to be modulated by the target/error likelihood (Polich, 1990; Polich and Margala, 1997; Jessup et al., 2010; Hauser et al., 2014). In conclusion, although ErrPs are modulated by the frequency of the stimulus they cannot be explained by this factor alone and seem more correlated to their meaning.

Another factor that can modulate the amplitude of the ErrP concerns the attention level of the subject and her/his engagement in the task (Hajcak et al., 2005). Subjects tend to have smaller ErrP amplitude when simply monitoring the device than when they are controlling it, c.f. Figures 2C,D (Ferrez and Millán, 2008a; Chavarriaga and Millán, 2010). This factor may influence the performance and, as with other BMI approaches, calls for efficient calibration methods, which could be used before online operation.

Overall, initial ErrP studies supported the idea that it was possible to identify erroneous responses–either manual or decoded through a BMI–and use them to correct these errors to improve the overall performance (c.f., Figure 1 Left). They were based on offline analysis and did not assess the effect of these approaches during online operation, but fostered continued efforts to reliably decode such error-related brain activity and to integrate it in the framework of human–machine interaction.

## 4. Error-related potentials as a corrective signal

### 4.1. Error correction in motor-related BMI

Subsequent attempts at integration of ErrP-based correction into online BMI setups yielded generally positive results. Extending their previous protocol, Ferrez and Millán (2008b) used a two-class MI-based BMI to control one-dimensional step-wise movements of a cursor. The potential evoked by the cursor movement was decoded to indicate an erroneous or correct movement. In the former case, the cursor was returned to the previous position. Simultaneous real-time decoding of both ErrPs and MI-related activity in two subjects resulted in a three-fold increase in the information transfer rate. They report 80% accuracy in the ErrP recognition, which lead to a reduction of the MI decoding error from about 30% to less than 9%. Kreilinger et al. (2009) also reported performance improvement in a similar experimental protocol involving 13 healthy subjects. MI classification accuracy increased from about 70 to 80% using the online ErrP-based correction.

Other studies have provided further support to the feasibility of using such hybrid approaches, combining the use of one BMI signal to decode the action commands and the ErrP decoding to correct erroneous actions. For instance, Artusi et al. (2011) using offline analysis showed improvement in the decoding of movement-related potentials (i.e., preparatory EEG activity before actual movement performance) by introducing ErrP classification. In their approach, the outcome of the movement decoder was shown to the user and if the elicited EEG response was decoded as corresponding to the error condition, the trial was discarded and the task had to be repeated. Their experiment, involving six healthy subjects, yielded an average ErrP recognition of 80%. Simulation of this corrective mechanism showed a reduction of the global error rate in discriminating between imagination of slow and fast arm flexions from 26% to 14%. In this case, 20% of the trials were discarded based on the ErrP decoding. They estimated an improvement in the average information transfer rate of 76%. Altogether these results are indeed encouraging; however, often such studies used simulated initial BMI commands in order to keep a constant performance; e.g., Ferrez and Millán (2008a); Millán et al. (2009); Artusi et al. (2011). The purpose of such manipulation is to decouple the estimation of the benefits of ErrP-based detection from within-session variations of the command decoder. In consequence, further online tests are required to fully assess the actual performance of motor-related BMIs combined with ErrP-triggered corrective actions.

### 4.2. Error correction in P300-based BMI

ErrP-based correction mechanisms have also been applied widely to P300-based spellers (Dal Seno et al., 2010; Takahashi et al., 2010; Combaz et al., 2012; Spüler et al., 2012; Schmidt et al., 2012). These systems exploit an event-related potential elicited by a rare, relevant stimulus: the so-called P300 ERP component (Farwell and Donchin, 1988). In this application, the interface can cancel a character selected with the P300-based speller upon subsequent ErrP detection or, alternatively, correct it by choosing the second most probable character according to the P300 decoding. Although, an early study showed little or no improvement by ErrP-based online correction for two subjects using a pseudorandom matrix speller (Dal Seno et al., 2010), later works showed advantage of integrating the ErrP detection into the BMI. Schmidt et al., (2012) reported an average increase of 40% in the writing speed of twelve healthy subjects using a speller interface designed to reduce the performance sensitivity to gaze shifts (Treder et al., 2011). More recently, Spüler et al. (2012) reported experiments with six subjects with motor disabilities (5 diagnosed with amyotrophic lateral sclerosis, ALS, and one with Duchenne muscular dystrophy) in which a performance increase was observed (0.37 bits per trial). For comparison, an age-matched group of eight able-bodied subjects showed an increase of 0.73 bits per trial, while a group of nine younger subjects had an increase of 0.44 bits per trial. Notably, patients with ALS exhibited similar ErrP patterns to those of healthy subjects, further supporting the potential use of error processing signals in such BMI applications, primarily meant for users with severe disabilities. Both studies found an inverse correlation between the performance improvement and the accuracy of the P300 decoder. However, conversely, a different study involving 16 healthy subjects reported larger improvement for users with higher spelling accuracy (Perrin et al., 2012). In this study, such subjects also showed a slightly higher specificity in the decoding of the ErrP signal. A potential issue to be taken into account in this approach is that both P300 and ErrPs are modulated by attentional processes (Yeung et al., 2005; Kleih et al., 2010). Therefore, factors that affect the level of engagement or motivation of the user (e.g., a BMI low accuracy, high mental workload) may be reflected in the elicited ERPs and, depending on the sensitivity of the decoder to these variations, be detrimental to the overall performance after integration of the ErrP-based correction in both able-bodied and users with disabilities.

### 4.3. Correction of manual responses

Besides using ErrPs to correct commands generated by BMI systems, these signals can also be used in HCI applications requiring manual responses from the user. The first attempts to decode the ERN/Pe components date back to the early 2000s. Blankertz et al., (2003) reported decoding of error-related signals in eight able-bodied subjects using a modified d-2 attention task (Bates and Lemay, 2004). Then, Parra et al. (2003) analyzed ErrPs in a forced choice visual discrimination task, i.e., the Eriksen Flankers task. They reported single-trial classification accuracy of 91% averaged over seven healthy subjects. Furthermore, online correction of the manual responses using the decoded error-related EEG correlates reduced the discrimination error rate in 5 of the subjects (average error reduction was 21.4 ± 21.7%). While in the above study users responded by pressing a key, Ventouras et al. (2011) also tested the decoding of the ERN/Pe in the Eriksen task using a joystick as input device. Their experiment included 16 healthy subjects, and classification performance was assessed using the leave-one-out procedure. They reported sensitivity and specificity values over 87.5%.

These signals have also been tested as a means to correct errors in typewriting tasks. Wang et al. (2011) decoded ErrPs elicited during a hear-and-type task where nine subjects had to type numbers dictated by a computer. They reported sensitivity and specificity values of 68.72 and 51.68% for classifiers trained and tested on the same subject. The performance for cross-subjects classifiers was 68.72 and 49.45%, respectively. A limitation of this study, and a potential reason for the low decoding performance, is the small number of keystroke errors made by the subjects, ranging from 0.42 to 3.58%. As discussed below, this limits the possibility of building proper models of the signals corresponding to errors.

Another interesting study evaluated both feedback and self-generated errors in a task involving visuo-tactile stimuli (Lehne et al., 2009). Eleven participants took part in the study where an array of vibrotactile stimulators provided information about a tactile cursor that should be directed toward a target location on the torso. Visual stimulus presented an intended direction of movement and upon its appearance, the user pressed a button to confirm or reject the proposed movement direction. Given the task difficulty, users made erroneous responses in 27.8% of the trials on average. Furthermore, in other trials machine errors were also introduced (i.e., the machine misinterpreted the button responses). Classification of both types of errors yielded accuracies of about 70%, with higher detection rates for the correct than the error trials (i.e., about 70 and 50%, respectively).

Overall, these works show the feasibility of decoding error-related information after user overt responses. In general the classification performance was higher for the correct condition, in particular when the complexity of the task increases.

## 5. Error-driven learning

The studies presented above used ErrP detection to immediately correct erroneous decisions made by the BMI. An alternative use of these signals is error-driven learning. This approach, illustrated in Figure 1 Right, has been applied to endow BMI systems with adaptive capabilities in two different manners. One possibility is to update the BMI classifier (Blumberg et al., 2007; Llera et al., 2011, 2012; Roset et al., 2013). For instance, Llera et al. (2011) used the decoding of error-related MEG activity to identify misclassification in a two-class covert visual attention paradigm (*N* = 8). The lateralization of alpha-band power in posterior channels was classified using logistic regression to infer which direction (i.e., left or right) the subject was covertly attending to. ErrP decoding was used to identify misclassifications and provide new labels for the incoming data in a semi-supervised manner. The labeled sample was then used to update the classifier parameters. Offline analysis showed that this approach can significantly increase the performance of the BMI classifier. Importantly, given that this is a binary task the intended target class can be easily inferred for the misclassified samples allowing the use of supervised learning techniques for the classifier adaptation. A similar strategy was adopted by Artusi et al. (2011) in the task described in section 4.1, i.e., decoding of imagery of fast vs slow movements. In their case, those trials that were considered as correct after ErrP classification were incorporated into a learning set that was used to perform online retraining of the MI classifier.

However, as long as the ErrP decoding is considered to be in essence binary, the overall performance will be substantially affected by the false positive rate (i.e., correct BMI actions misclassified as errors). A way to palliate this effect is to use methods relying on probabilistic error signals. In that way the reliability of the ErrP decoder (estimated from the training set) can be taken into account. Bayesian filtering or Expectation-Maximization have been put forward as possible approaches (Perrin et al., 2010; Artusi et al., 2011; Llera et al., 2012). A similar method was also proposed in a hybrid system for human-computer interaction where an acceleration-based gesture recognition system was updated using the decoding of the ErrP signal (Chavarriaga et al., 2010). However, as per our knowledge they have only been tested in offline experiments.

Besides adapting the BMI classifier, ErrPs can be used to improve the behavior of a semi-autonomous system. This approach is anchored in the concept of shared control, where intelligent devices can take care of low-level decisions while the user only provides high-level commands –using a BMI or another input modality– (Perrin et al., 2010). In this case, the user monitors the performance of the intelligent device and whenever an ErrP is detected, suggesting the action was perceived as erroneous, it is used to adapt the device controller to reduce the likelihood of committing the same error later on (Chavarriaga and Millán, 2010). In terms of the reinforcement learning algorithm, the detection of an ErrP corresponding to the error condition will be translated onto a negative reward value, effectively punishing the performed action when updating the control policy. This approach was first tested in a 1-D control task with six healthy subjects (c.f. Figure 3A). The offline analysis showed that it was possible for the device to learn optimal control policies even though the accuracy of the ErrP decoder was not perfect. An online evaluation of this approach on two subjects monitoring a simulated robot was demonstrated by Iturrate et al. (2010). In this work the subject had to choose the intended target location of the robot and then monitor its movements. The decoded ErrPs were used in a reinforcement learning paradigm to update the robot control policy. They reported that the learned policy converged toward the optimal one–i.e., taking the robot to the user's intended location–in 92% and 75% of the cases for each subject, respectively. Recent integration with shared control techniques suggests that further improvements in performance can be achieved (Iturrate et al., 2013b). In this case 4 subjects monitored a moving cursor in a 2D reaching task, and the ErrPs were used to select one control policy from a pre-defined repertoire; i.e., selecting the most suitable policy to reach the inferred target location.

## 6. Error-related potentials in realistic applications

As summarized in section 2, a wealth of neuroscience literature has reported error-related neural correlates. These studies are typically performed in well-controlled laboratory conditions using abstract tasks and stimuli. This allows characterization of such correlates in recording conditions that yield higher signal-to-noise ratio and avoid confounds that may appear when allowing more behavioral freedom to the subject(user), or relaxing constraints of the operational setting.

Notably, several studies presented in previous sections corroborated the existence of similar correlates during complex scenarios or realistic interaction with complex devices. Wang et al. (2011) evaluated ErrPs when users performed a typewriting task, while Spüler et al. (2012); Schmidt et al., (2012) and others have tested these potentials while subjects use a P300-based speller. Moreover, it has been possible to observe and decode error-related potentials while people monitor the performance of a robotic arm (Kreilinger et al., 2012; Iturrate et al., 2014) or a mobile robot (Perrin et al., 2010; Chavarriaga et al., 2012), both using simulated and real platforms. Similar correlates were also found during simulated driving of an intelligent car (Zhang et al., 2013). Another study, assessing potential BMI applications to cope with the situational disability experienced by astronauts, reported similar ErrP waveforms and decoding performance under different gravity conditions in parabolic flights (Millán et al., 2009).

These studies suggest that these ErrPs can also be decoded in more complex tasks and scenarios. Nevertheless, it has to be noticed that the decoding performance is typically lower than in simpler, well-controlled experimental paradigms. The performance differences can be due to the decreased signal to noise ratio of the recorded signals, as well as the increased workload placed on the user in the complex tasks. As shown below, some works have attempted to identify and exploit common patterns between simple and complex tasks as a procedure to improve the training of the ErrP decoder in more challenging conditions (Kim and Kirchner, 2013; Iturrate et al., 2014).

## 7. Classification of error-related potentials

A key factor for exploiting ErrPs to improve BMI performance is the ability to decode this signal in a single-trial. As it is the case for all BMI systems, they rely on the real-time processing of the neural signals and the use of machine learning techniques to relate the current activity pattern to a corresponding class (i.e., error or correct condition). This process involves the extraction of suitable features and the training of a classifier based on available labeled samples. Below we discuss the most common methods applied for decoding the error correlates. However, a comprehensive review of the machine learning methods applied in BMI is out of the scope of this paper. Interested readers can refer, among others, to introductory papers by Bashashati et al., (2007); Lotte et al., (2007) and Blankertz et al., (2011).

Studies presented in the previous sections show that it is possible to decode the ErrPs. Noticeably, they have often reported higher classification accuracy for correct trials than errors. This may be partly due to the protocols used to train the classifier. These typically involve a low error-rate (e.g., 20%) thus yielding a larger number of examples for the correct class. The use of an imbalanced number of samples per class may result in asymmetric costs for misclassification of each class and leads to classifiers that are biased toward one of the classes. Moreover, it is difficult to properly estimate the classifier parameters if only a limited number of examples is available.

Regarding the processing and classification techniques used to decode the ErrPs it can be observed that a vast majority of the reported studies are based on temporal features (i.e., waveform shape) computed from a few pre-selected electrodes in the fronto-central areas (e.g., FCz, Cz). Typically, EEG signals were low-pass filtered below 10 or 20 Hz and time-samples from a pre-defined window (usually between 200 and 600 ms) were used for classification (Blankertz et al., 2003; Ferrez and Millán, 2008a; Kreilinger et al., 2009; Chavarriaga and Millán, 2010; Dal Seno et al., 2010; Takahashi et al., 2010; Artusi et al., 2011; Spüler et al., 2012). In some cases, authors used automatic selection mechanisms over larger feature spaces, quantifying discriminant power of features with some metric, e.g., t-statistic, Fisher score or *r*^2^ (Dal Seno et al., 2010; Goel et al., 2011; Iturrate et al., 2013a). These studies reported similar features than those manually selected but aimed at better capturing subject-dependent variations in the elicited signals. Alternative approaches to compute features have recently been proposed including the usage of spatiotemporal filters (Perrin et al., 2012; Rousseau et al., 2012; Iturrate et al., 2013a, 2014), as well as singular value decomposition (Hamner et al., 2011; Phlypo et al., 2011).

A few studies have tested the feasibility of exploiting features computed in the frequency domain (Bollon et al., 2009; Omedes et al., 2013) with generally encouraging results. Interestingly, Omedes et al., (2013) tested the use of theta power as a feature for classification in the three experiments shown in Figure 3. They evaluated which type of features generalize better across tasks by measuring the classifier performance in a different task than the one it was trained for. Offline tests of data from six subjects showed smaller performance degradation across tasks for classifiers using frequency features compared to those using temporal features. A separate study showed that ErrP variation across these tasks is mainly due to latency (Iturrate et al., 2014). This suggests that frequency-based features may be less sensitive to temporal jitter across individual ErrP trials.

Goel et al. (2011) tested the use of features computed on the intracranial EEG sources, which have been estimated using inverse solution methods. The hypothesis being that projection into the source space can act as a spatial filtering technique that increases the signal-to-noise ratio of neurophysiologically relevant discriminant features. Offline analysis in the monitoring protocol depicted in Figure 3A, showed improved performance–in terms of area under the curve, AUC (Fawcett, 2006)–with respect to previously reported results using surface EEG classifiers in the six subjects analyzed. Further online experiments confirmed the validity of using these features for classification although its potential advantages over standard methods is yet to be fully validated (Goel, 2013).

Taking into consideration the cross-regional interactions reported in neurophysiological studies (c.f., section 2), Zhang et al. (2012) evaluated the use of single-trial estimation of connectivity patterns for ErrP classification. They computed directional interaction across channels using a modified directed transfer function (DTF) method in different frequency bands (Kamiński and Blinowska, 1991). Offline analysis of data on 16 subjects using the same monitoring protocol as above, showed discriminant fronto-central interactions in the theta rhythm that yielded single-trial decoding above chance level. Furthermore, the combined use of connectivity and time-based features gave significantly better performance than temporal features alone, suggesting that the two feature sets convey complementary information.

The most common classification techniques used for the decoding include linear discriminant analysis (LDA) or its variations such as Fisher LDA or regularized LDA (Blankertz et al., 2003; Parra et al., 2003; Lehne et al., 2009; Ventouras et al., 2011; Iturrate et al., 2014), as well as Gaussian classifiers (Ferrez and Millán, 2008a; Kreilinger et al., 2009; Chavarriaga and Millán, 2010; Perrin et al., 2012) and support-vector machines (SVM) (Artusi et al., 2011; Ventouras et al., 2011; Wang et al., 2011; Spüler et al., 2012). In their study, Spüler et al. (2012) performed an offline comparison of LDA, step-wise LDA, and SVMs with linear and radial basis function (RBF) kernels. For this analysis they used previously recorded data of six patients with ALS. Using 10-fold cross-validation test they selected the RBF-kernel SVM as the best suitable for their application (P300-speller). Unfortunately, they did not report what specific criteria were used for this selection nor the performance of each method. Similarly, Ventouras et al. (2011) compared the performance of SVM and K-NN classifiers on the decoding of ErrPs elicited after manual responses. Their analysis using different feature selection mechanisms and leave-one-out cross-validation showed no significant differences between the two methods. Wang et al. (2011) also compared classifier performance when decoding those signals. Interestingly, they found little performance differences between the sensitivity obtained with LDA and SVM classifiers when training and testing on the same subject's data. In contrast, when the test was performed on a subject that was not part of the training set, the LDA yielded higher sensitivity. However, their results were close to chance level and may not be significant given that only a small number of trials were available for the error class.

A direct comparison of the performance obtained in these studies cannot be interpreted as a fair assessment of the advantages of a given decoding method. That is due to the differences in the pre-processing methods applied, the features selected for classification, and the reported performance metrics. Nevertheless, they often reported classification accuracies between 70 and 80%. As a tentative synthesis, one is under the impression that various classification methods reported in literature seem to obtain comparable results. Taking into account that most of these studies involve a rather small number of subjects, it is very likely that any performance differences are largely influenced by inter-subject variability.

In addition, efforts have been undertaken to design methods for fast training of the ErrP decoder. Some recent approaches rely on semi-supervised or unsupervised learning (Grizou et al., 2014; Zeyl and Chau, 2014). Another applied technique is the use of available data from other subjects to boost learning of a subject-dependent classifier (Iturrate et al., 2011; Putze et al., 2013). Alternatively, ErrPs have been shown to have common characteristics across tasks. In consequence, several methods have been proposed for online adaptation of classifiers trained in a previous protocol to the characteristics of the potentials elicited in the new task (Iturrate et al., 2013a; Kim and Kirchner, 2013; Iturrate et al., 2014). In this case, provided that an ErrP decoder has already been trained in a given task, the calibration time for a new task can be considerably reduced. Finally, as mentioned above, frequency features seemed less sensitive to task-dependent latency jitters in the neural response and were thus proposed as a potential means to implement task-independent classifiers (Omedes et al., 2013). These techniques have shown encouraging results, but have yet to be thoroughly tested to confirm their real advantages.

Lastly, besides direct single-trial classification problems, the ErrP detection process should take into account how this information will be later on utilized for interaction. In particular, there may be application-dependent requirements in terms of sensitivity and specificity that need to be considered when choosing the classification technique and parameters (Parra et al., 2003; Seno et al., 2010; Spüler et al., 2012).

## 8. Discussion

The works reviewed in this paper strongly support the feasibility of decoding error-related potentials and using the information they carry to improve the performance of BMI and HCI systems. There are, however, several challenges that need to be overcome until efficient and fully working applications can be implemented in the real world.

First of all, there is a clear need for further evaluation of the online exploitation of ErrPs. Although there is an increasing amount of studies showing online decoding of error-related signals, in particular for P300 applications, they typically rely on a small number of subjects. These studies have already highlighted how individual differences may affect the overall performance of the error-correction mechanism (Perrin et al., 2012; Schmidt et al., 2012; Spüler et al., 2012). Therefore caution should be taken in the design of such studies. In particular, this includes the number of subjects involved in the study, the control conditions against which the performance will be evaluated, as well as the effects of subject learning and fatigue when testing over several sessions.

Further studies are also needed to evaluate the detection of these potentials in people with severe disabilities, which is the principal target user group of BMI today (one of the main potential applications of BMIs is the restoration or substitution of motor and communication capabilities). Spüler et al. (2012) reported encouraging results, showing that reliable ErrPs are elicited in patients with ALS and their decoding can improve performance of a P300-speller. Nonetheless, there is clearly a need for more studies characterizing these signals in different populations. Some studies have already pointed out age-related changes in the ERN (Davies et al., 2004; Wiersema et al., 2007), but it is yet to be assessed how these changes affect the discriminability between error and correct trials. Similarly, longitudinal studies may be necessary to identify how ErrPs change in the case of degenerative diseases. In addition, several works have pointed out that feedback modalities other than visual may be suitable for users in the locked-in state as they do not rely on volitional gaze control (Schreuder et al., 2010; Treder et al., 2011; Kaufmann et al., 2013). Preliminary evidence suggests that ErrPs can be elicited and, to some extent, decoded after tactile stimulation (Lehne et al., 2009; Chavarriaga et al., 2012) in healthy users. This possibility remains to be further tested, in particular in users with disabilities.

Another issue of interest concerns the evaluation of the performance of hybrid BMI systems exploiting ErrP-decoding. Typically, authors have reported changes using diverse metrics including accuracy, information transfer rate (Wolpaw et al., 2000), efficiency (Quitadamo et al., 2012) or utility value (Seno et al., 2010). This denotes a lack of a formal framework for performance assessment –a problem common to the overall BMI field– and prevents the comparison of results across different studies (Thomas et al., 2013; Thompson et al., 2014). It is advisable that future works provide a comprehensive evaluation of performance reporting different metrics to enable such comparisons. Moreover, performance can be affected by protocol-specific parameters. For instance, in the case of ErrP-based correction, each command correction will have a cost (e.g., time required to undo the last action), and the overall benefit of the correction mechanism will depend on both: this cost and the specificity of the ErrP decoder (Parra et al., 2003).

This is intrinsically linked to the sensitivity and specificity of the decoding performance. The impact of the falsely decoded trials will be highly dependent on the application and the actions taken upon error detection. A clear difference is observed between the corrective and learning use of the ErrPs. In the first case, ErrP classification errors are explicitly perceived by the user. This can be counterproductive if the false detections appear to impair proper use of the interface (e.g., by rejecting or changing correct commands), even if improved performance is achieved in the long-term. As an example, Perrin et al., (2012) reported that some users, despite having good ErrP decoding performance, still preferred the implementation of the P300 speller without the correction, since they perceived no benefit with respect to use of the P300 alone.

In contrast, the learning approach where the classifier or the device controller is updated according to the outcome of the ErrP decoding may mask these false detections. Moreover, it has been shown that reinforcement learning algorithms can converge toward optimal policies even in the case of noisy estimation of the reward signals, provided that the estimation performance is above chance level (Sutton and Barto, 1998). One can expect, thus, that the use of ErrPs for learning has lower requirements in terms of the minimal acceptable performance than immediate command correction. This holds, of course, provided that the initial performance of the control interface is already acceptable for the user. Future work assessing these requirements from a human factors perspective is certainly needed for effectively designing interaction systems that exploit these error-related signals.

Following basic studies on the ERN and FRN, BMI efforts of decoding error-related signals have so far mostly focused on the time-locked response generated by a discrete feedback stimulus. In consequence, exploitation of the ErrPs has been largely restricted to discrete tasks such as the P300-based speller or step-wise movements (c.f. section 3). Besides limiting the range of applications and their naturalness, this approach also limits the throughput of the system since after each command a time interval of several hundred milliseconds is required for evaluating the presence of an ErrP. Consequently, further research is required toward decoding in more continuous setups.

Although not an easy task, several lines of progress seem to be open. One alternative is to increase the pace at which the stimuli are presented. Typically, experiments exploiting ErrPs have an inter-stimulus interval of 2 s or more. In contrast, experiments using rapid serial visual presentation (RSVP) have shown single-trial decoding of EEG correlates of visual recognition with stimulus presentation rates higher than 4 Hz (Gerson et al., 2006). It still to be tested whether ErrPs can be detected in such fast-paced feedback presentations.

Another approach is combining usage of continuous feedback with additional discrete sensory events, used to time-lock ErrPs (Kreilinger et al., 2011, 2012). The first study used a game application where the decoding of MI-related patterns controlled lane changes in a continuously moving animated car. It provided feedback for correct or wrong changes in the form of multiple predictable collisions with point tokens (positioned on the correct lane) or barriers (on the wrong lane). The second work applied an interesting approach to the BMI-control of a robot arm, combining the performance of a continuous mental task with discrete feedback to elicit ErrPs. Subjects had to perform MI during a given period, then the robot arm moved and after that users should assess whether the robot's movement lasted the same amount of time as the MI task. Visual cues provided information of the robot movement duration. Offline analysis showed ErrP decoding above random level; however the performance in both studies was lower than those reported in purely discrete paradigms.

Besides the previous approaches employing, in essence, some strategy of circumvention of the continuous feedback problem, one can also try to directly tackle the possibility of decoding error-related signals in a purely asynchronous (non-time locked) manner. An example of such attempt with invasive electrocorticographic recordings (ECoG), benefiting from better signal-to-noise ratio than scalp EEG, demonstrated that error events can potentially be detected with good accuracy during a continuous task, given a temporal tolerance of several hundred milliseconds (Milekovic et al., 2013). For EEG, a potential avenue to explore is the use of the spectral content instead of temporal features. As shown in Figure 2 the event-related ErrPs are characterized by positive theta modulations. Interestingly, it has been shown that erroneous manual responses elicit increases in both phase- and non-phase-locked theta activity (Trujillo and Allen, 2007). Moreover, the power increase in non-phase locked activity was higher than for the phase-locked activity. Noticeably, ErrP-decoding performance based on theta-power features was shown to be less sensitive to task changes (Omedes et al., 2013). As already mentioned the main ErrP changes across these tasks can be explained by latency shifts (Iturrate et al., 2014). Thus supporting the notion that oscillatory activity can allow asynchronous detection of erroneous events in continuous tasks.

To summarize, different studies have repeatedly demonstrated the feasibility of decoding error-related EEG signals on a single-trial basis. This has been achieved both when the errors are committed by the user, as well as when the errors are introduced by the interfacing device, in particular a BMI. The decoding accuracy of these signals is typically about 80%. This performance levels have been shown to usually be sufficient to improve the information transfer rate in different applications including motor-imagery based BMIs, P300 spelling and manual labeling of visual stimuli (c.f. section 4). Moreover, ErrPs can successfully be used as a learning signal to improve BMI decoders or the controller of an external device (c.f., section 5). All these results support the potential of error-related correlates to provide naturally elicited information about the user cognitive state that can be used to adjust the machine's behavior.

Despite these successful studies, several aspects remain to be further explored, as detailed above. These include improvement in decoding of these signals in more complex applications, as well as their further characterization in subjects with disabilities. More importantly, large scale evaluations involving end-users have to be performed from a user-centered perspective to identify performance requirements and design criteria that allow for optimal exploitation of these correlates in practical applications.

## Funding

This work has been supported by the Swiss-funded SNSF NCCR Robotics. This paper only reflects the authors' view and funding agencies are not liable for any use that may be made of the information contained herein.

### Conflict of interest statement

The authors declare that the research was conducted in the absence of any commercial or financial relationships that could be construed as a potential conflict of interest.
